# German and Korean mothers' sensitivity and related parenting beliefs

**DOI:** 10.3389/fpsyg.2013.00561

**Published:** 2013-08-27

**Authors:** Jeanette Ziehm, Gisela Trommsdorff, Tobias Heikamp, Seong-Yeon Park

**Affiliations:** ^1^Department of Psychology, Developmental Psychology and Cross-Cultural Psychology, University of KonstanzKonstanz, Germany; ^2^Department of Child Development, College of Social Science, Ewha Womans UniversitySeoul, South Korea

**Keywords:** sensitivity, parenting, socialization, culture, Germany, South Korea

## Abstract

This study contributes to a differentiated understanding of maternal sensitivity in cultural and situational context. We investigated differences and similarities in German and Korean mothers' maternal sensitivity. We interviewed 92 German and 100 Korean mothers of first graders about their preference for proactive (anticipating children's needs) or reactive sensitivity (responding to children's direct cues) in different scenarios. Related parenting beliefs were assessed by asking the mothers to explain the reasons why they would prefer specific parenting behaviors. Results revealed significant cultural differences in reactive vs. proactive sensitivity preferences. Overall, German mothers were more likely to indicate that a mother should respond reactively and less likely to report that a mother should act proactively than were Korean mothers. Korean mothers gave preference to both reactive and proactive sensitivity depending on the scenario. With regard to parenting beliefs, analyses revealed that German and Korean mothers who preferred reactive sensitivity mainly explained their choices as attempts to encourage children's development of independence. In contrast, Korean and German mothers with a preference for proactive sensitivity were more likely to report that mothers would assist their children due to their immaturity in dealing with emotional distress. Results are discussed in the framework of the different meanings and functions of maternal sensitivity for socialization in different cultural contexts.

Mental beliefs are affected by intuitive theories that are shaped by cultural values. These theories are implicit and influence behavior and social interactions (Gelman and Legare, 2011). Parental intuitive theories [also referred to as ethnotheories, cultural belief systems, e.g., Harkness and Super (2006), or naïve theories, e.g., Kornadt and Trommsdorff (1990)] are cultural models of parents regarding children's development, family, and the self as a parent (Harkness and Super, 2006). Parental intuitive theories are part of the developmental niche, which includes three interacting subsystems: (a) the physical and social settings in which the child lives, (b) child care practices, and (c) the psychology of the caregivers. The latter subsystem involves parents' intuitive theories (Super and Harkness, 1997; Harkness and Super, 2006). Parents usually try to foster children's well-being by pursuing goals and respective parenting behaviors that will improve children's developmental outcomes and cultural fit (Bornstein, 1991; Trommsdorff, in press a). However, by which behavior a specific goal can be accomplished differs according to the caregiver's intuitive theories, which are influenced by value orientations and self-construals (i.e., independence, interdependence; Harkness and Super, 1996; Bornstein and Cheah, 2006; Trommsdorff, in press a).

We do not consider different cultures to be homogenous entities. Therefore, dichotomization of cultures according to theoretical concepts like individualism and collectivism are not sufficient to describe the dynamic and changing nature of socio-cultural contexts of development. According to Oyserman et al. (2002) both individualistic and collectivistic values are internalized by people in every cultural context to varying degrees. Nevertheless, Oyserman and colleagues concluded that it is worth saving the concepts of collectivism and individualism. Culture informed research has suggested that in contexts in which individualism is emphasized, parents pursue children's independence as an important socialization goal and highly value individuality and self-expression. On the other hand, children's dedication to their family and social in-groups is a crucial socialization goal for parents living in cultures in which interdependence is emphasized because group harmony and self-restraint are highly valued (Rothbaum and Trommsdorff, 2007; Rothbaum and Wang, 2010).

Maternal sensitivity is a specific kind of parenting behavior that is influenced by culture-specific intuitive theories. The aim of this study is to investigate differences and similarities with regard to German and South Korean mothers' sensitivity and related parenting beliefs. Here, parenting beliefs are defined as intentions that motivate specific parenting behaviors, namely proactive and reactive sensitivity (Trommsdorff et al., 2012).

## Maternal sensitivity

According to attachment research, maternal sensitivity is linked to several positive developmental outcomes for children (e.g., social and emotional competence; Bowlby, 1969; Ainsworth et al., 1978; Bornstein and Tamis--LeMonda, 1989; Belsky and Fearon, 2002; Thompson, 2008). Grusec and Davidov (2010) suggested that socialization takes place in different “domains” requiring specific parenting behaviors to achieve particular socialization goals. For studying maternal sensitivity, the domain of protection (i.e., caregiver–child interactions in which a caregiver provides comfort and security to a child) is of special relevance. This domain requires parenting behavior that helps children to alleviate their distress.

Ainsworth et al. (1978) defined sensitivity as prompt and appropriate maternal responses to children's signals, which, according to the sensitivity hypothesis, is the basis for children's secure attachment (e.g., Grossmann et al., 2005). Rothbaum and colleagues, however, questioned the universality of this hypothesis by referring to cross-cultural differences with regard to the expression of sensitivity and its function for development (Rothbaum et al., 2000). Indeed, recent research has pointed out the prevalence of cultural differences in the meaning of sensitivity for parent–child relationships in different cultural and situational contexts and developmental stages (e.g., Bornstein et al., 1992; Richman et al., 1992; Keller et al., 2002; Rothbaum et al., 2006; Trommsdorff and Friedlmeier, 2010). In accordance with these findings, it is suggested that caregivers' sensitivity in Western cultures can be described as reactive. In contrast, caregivers' sensitivity in non-Western cultures, in which children's interdependence is highly valued, can be characterized as proactive (Trommsdorff and Rothbaum, 2008). Reactive sensitivity means that caregivers respond to children's direct signals, hence caregivers expect children to express their needs explicitly. Proactive sensitivity can be understood as anticipating children's needs by observing and interpreting the children's behavior. Therefore, children are not expected to explicitly communicate their needs (Rothbaum et al., 2006; Trommsdorff and Rothbaum, 2008).

### Parenting beliefs related to different forms of sensitivity

Parenting beliefs are parents' ideas about children which influence parenting behavior (e.g., maternal sensitivity) in order to achieve desirable and to avoid undesirable developmental outcomes and to promote children's well-being (Super and Harkness, 1997). Albeit, by which behavior a specific outcome can be accomplished differs according to the caregiver's parenting beliefs which we assume to be influenced by the cultural context (Trommsdorff, in press a). In cultures in which autonomy is emphasized children's independence is believed to be very important because individuality and self-expression are highly valued. On the other hand, children's relatedness to their family and social in-groups is crucial in cultures in which interdependence is emphasized because group harmony and self-restraint are highly esteemed attributes (Rothbaum and Trommsdorff, 2007). Therefore, it is assumed that sensitivity in Western contexts is related to the support of children's exploration and autonomy and that mothers' sensitivity in non-Western cultures aims more to establish dependency and emotional closeness (Rothbaum et al., 2000, 2006).

### A closer look at cultural differences in sensitivity

Maternal sensitivity can be expressed in different ways depending on the underlying parenting beliefs. For example, Trommsdorff and Friedlmeier (2010) induced preschool girls' negative emotions and found that German mothers intervened only after their children expressed distress. In contrast, Japanese mothers responded to their children before their distress was fully expressed. According to the authors, these effects can be interpreted in terms of different underlying parenting goals concerning the socialization of children's emotion regulation (authentic expression vs. suppression of emotions). Further, the Japanese mothers' sensitivity varied according to the situational context whereas there were no changes in the sensitivity of the German mothers. The authors concluded that Japanese mothers' sensitivity could be seen as more flexible and situation specific, whereas German mothers' sensitivity was more stable across situations (Trommsdorff and Friedlmeier, 2010).

In their study on cultural differences in caregivers' beliefs about sensitivity, Rothbaum et al. (2006) asked Japanese and American teachers about their preference for different forms of sensitivity in various scenarios: they could either anticipate children's needs or respond to children's direct requests. American teachers were more likely to respond to direct signals of their students. They reasoned that children should learn to rely on themselves and take the responsibility for satisfying their needs. Furthermore, the American teachers were also more likely to report that they aimed to foster children's self-expression. The Japanese teachers, who preferred to anticipate children's needs, felt to be responsible for correctly anticipating and fulfilling children's needs in order to promote children's reliance on their teachers (Rothbaum et al., 2006). Thus, these findings suggested cultural differences in caregivers' expression of sensitivity.

## The present study

In the present study, we investigated similarities and differences between German and Korean mothers of first graders regarding their sensitivity and related parenting beliefs across varying situations. We chose a comparison between mothers from Germany and mothers from South Korea because we assume that maternal sensitivity differs according to the cultural context. German mothers, living in a Western context, are expected to explain their behavior as encouraging children's independence, whereas Korean mothers, from a collectivistic-oriented country, are presumed to highly value relatedness, interdependence, and social harmony as socialization goals (Hofstede, 1980; Kim et al., 2005; Schwarz et al., 2005; Choi et al., 2013).

In line with Raeff (2010), we conceive of cultural contexts as dynamic systems of social interaction in which multiple value orientations coexist that shape caregivers' beliefs about parenting, parenting intentions, and related behavior. We also acknowledge that there is considerable within-cultural variability regarding caregivers' aims of fostering rather independence or interdependence in their children.

Core values of Korean family socialization include family hierarchy, demonstration of respect, maintaining appropriate etiquette with parents, family obligations and ties, achievement orientation, and strict parenting styles (Choi et al., 2013). Korean parents' sensitivity is characterized by monitoring children's behavior constantly and speaking for their children. The relationship between mother and child is characterized by a very close mother–child bond. For instance, the Korean concept of *hyo* stands for filial piety and is seen as an important component in building positive parent–child relationships (e.g., Kim, 2006), whereas caregivers in Western cultures perceive their children as independent individuals (Choi, 1992; Rothbaum et al., 2006).

However, in contemporary South Korea, the value of autonomy in children is rising. This might be related to an enormous and continuous growth of economic success and education for the last 50 years (Trommsdorff, in press b). Accordingly, social changes may affect socialization conditions. The number of traditional extended families and the fertility rate are on the decline, and the number of working mothers is increasing [see e.g., Kim et al. (2005), for an overview]. Due to those changes and the extended access to Western-based information (e.g., provided by literature, the Internet, and media) it is assumed that Korean caregivers' parenting is influenced by modernization and adaptation to Western parenting beliefs (Park and Cheah, 2005; Cheah and Park, 2006). Nonetheless, traditional Korean values continue to be preserved (Kim et al., 2005; Park and Cheah, 2005; Schwarz et al., 2005; Chang and Song, 2010; Choi et al., 2013).

In a recent study by Park and colleagues, Korean mothers reported that they would generally prefer both proactive as well as reactive sensitivity. When asked if mothers should always observe their children and if mothers should approach a sad child they were more likely to choose proactive sensitivity. The mothers explained this preference by referring to children's developmental stage and their goal of preventing accidents by intervening beforehand. In contrast, mothers who reported that they would expect their children to clearly communicate their needs (reactive sensitivity) explained that they aimed to encourage children's autonomy and independence (Park et al., 2012).

In the present study, we aimed to investigate whether or not German and Korean mothers differ in their maternal sensitivity preferences. As Rothbaum et al. (2006) note regarding proactive and reactive sensitivity “there is no direct evidence of cultural differences in caregivers (parents' or teachers') preference of the two types of sensitivity” (p. 27). The literature reviewed in the present article indicates that there may be differences in maternal sensitivity which are related to cultural values. Further, the articles cited refer mainly to socialization of infants and preschool children but not to children who recently entered school. The study by Rothbaum et al. (2006) was the first one that investigated cultural differences in pro- and reactive sensitivity. Since this was done by interviewing preschool teachers it is not clear whether these findings also hold for mothers of first graders. Moreover, the previous study focused on an American-Japanese comparison. A comparison between German and Korean mothers' preferences for different forms of sensitivity is new. Further, our study is the first one to analyze parenting beliefs related to pro- and reactive sensitivity in a cross-cultural comparison. As the study by Park et al. (2012) indicates both reactive as well as proactive sensitivity can be found in South Korean mothers. According to the notion that South Korea is subject to “Westernization” (e.g., Kim et al., 2005; Chang and Song, 2010) but that traditional Korean values still prevail (e.g., Park and Cheah, 2005; Schwarz et al., 2005; Chang and Song, 2010), it is fruitful to compare Korean mothers to mothers living in a Western context in order to elaborate the understanding of sensitivity in cultural context. Therefore, the objective of the present study is not only to investigate cultural differences but also to delineate similarities with regard to mothers' sensitivity and parenting beliefs. In particular, we expected (a) that both German and Korean mothers would aim to promote children's independence by encouraging their children to express their needs openly (reactive sensitivity) while this kind of sensitivity would be more common in the German sample; and (b) that Korean mothers would be more likely to prefer proactive sensitivity in order to foster emotionally close relationships.

## Methods

### Sample

The German participants were recruited through kindergartens, schools, and citizen registration offices in Southern Germany. The German sample consisted of 92 mothers, 48 (52%) of whom had sons and 44 (48%) of whom had daughters. The mothers were 41 years old on average (*M* = 40.86, *SD* = 4.49) and children were between 6 and 7 years old (*M* = 6.81, *SD* = 0.44). The Korean sample was recruited from public and private elementary schools in Seoul, South Korea. Overall, 100 mothers were interviewed. The average age of mothers was 36 years (*M* = 36.23, *SD* = 3.24). The children (55% boys and 45% girls) were 6–7 years old (*M* = 6.70, *SD* = 0.31).

The German and Korean samples differed significantly with respect to mothers' age *t*_(165)_ = −8.13, *p* < 0.001 and average number of children (Germany: *M* = 2.36, *SD* = 1.07; Korea: *M* = 1.94, *SD* = 0.53), *t*_(131)_ = −3.41, *p* < 0.01. Moreover, a higher percentage of German (72%) in comparison to Korean mothers (33%) were employed χ ^2^(1, *N* = 192) = 28.79, *p* < 0.001. The samples also differed according to mothers' vocational education χ ^2^(1, *N* = 192) = 76.59, *p* < 0.001. Here, the majority of the Korean mothers had a bachelor's degree (52%) or an accomplished apprenticeship (31%) (master's degree 11%, doctoral degree 3%, no vocational education 3%) and the majority of the German mothers had a completed vocational training (49%) followed by a master's degree (41%) (doctoral degree 4%, higher vocational education 3%, no vocational education 2%). German and Korean mothers did not differ regarding their socio-economic status *t*_(190)_ = −0.91, *p* = 0.364.

### Procedures

Mothers were interviewed by a trained member of the respective team either in their homes or at the respective university. Mothers' answers to forced-choice questions were written down by the interviewer. Open-ended questions were audio taped and transcribed by members of the respective team. The original interview was formulated in English, translated into the respective language of the participants, and back translated into English by native speakers. The back-translated interviews were compared to the originals and discrepancies were resolved by joint discussions between the translators and principal investigators from each country.

#### Maternal sensitivity

Maternal sensitivity was assessed with the Caregiver Sensitivity Interview (CSI, adapted from Rothbaum et al., 2006; see also Park et al., 2012; Trommsdorff et al., 2012). The original interview consisted of 12 scenarios designed to elicit beliefs about sensitivity in everyday situations. Five of the 12 scenarios, which were originally constructed for assessing teachers' preferences about anticipating or responding to children's needs in the school context, were chosen and slightly adjusted to fit mothers. For instance, references to the school context were deleted and culturally appropriate wording was administered [e.g., “if a child is in a bad mood” was changed to “if a child does not feel well happy”; see Rothbaum et al. (2006), for the original wording of items]. The interview consisted of five scenarios, and mothers judged how a mother or a child should behave and how the mother herself would behave in the specific situations. Therefore, the mothers provided information on their own role as a mother (normative as well as non-normative) and their expectations regarding the child's role. This was done in order to investigate differences and similarities regarding different aspects of maternal sensitivity (maternal attention toward the child, maternal reactions regarding children's behavior, maternal preferences regarding children's expression of needs). After the presentation of each scenario, the mother selected one of the two response alternatives (forced choice) with one option representing reactive sensitivity and the other representing proactive sensitivity.

In Scenario One participants were asked whether a mother should always observe a child in order to know when to offer help or wait until the child requests help. Scenario Two described a situation in which a child stumbled over a stone but did not cry. Here the mothers had to decide whether they would approach the child and comfort him/her or whether they would wait and see what happens and in case that the child starts crying they would come and comfort the child. Similarly, in Scenario Three, the mothers had to choose whether a mother should sit close to an obviously unhappy child or to let the child know that she/he could approach the mother if needed. In Scenario Four, the mothers were asked whether children should ask for help when needed or wait until the mother provides help. In Scenario Five, the mothers had to decide whether a mother should attend to children's explicit requests or anticipate children's needs.

#### Parenting beliefs

Parenting beliefs related to proactive and reactive sensitivity were assessed by asking the mothers to explain the reasons for their choice (“Could you tell me why you would think/do this way?”) after each scenario of the CSI. Mothers' responses to the open-ended questions were categorized according to a coding scheme developed by the fourth author of this article (2–6 categories for each proactive and reactive behavior for each scenario, e.g., reactive behavior: the child needs to learn what to do by her/himself or deal with problems independently; the mother cannot know everything that the child may need; proactive behavior: the child is too young to know what to do; the child needs someone who provides comfort).

German and Korean mothers' responses were coded by a German and a Korean rater, respectively. In order to assess interrater-reliability, 25% of cases (25 German as well as of 25 Korean mothers) were randomly selected and were coded by second raters (a German rater coded 25 German cases and a Korean rater coded 25 Korean cases). *Cohen*'*s Kappa* was above 0.75 and highly significant (*p* < 0.001) for each category.

## Results

Binary logistic regression analyses were computed to investigate cultural differences regarding sensitivity and parenting beliefs in each scenario. In all analyses we controlled for mothers' age, working status, and mothers' overall number of children to make sure that cultural differences are not implicit effects of demographic variables.

### Maternal sensitivity

**Figure 1:**
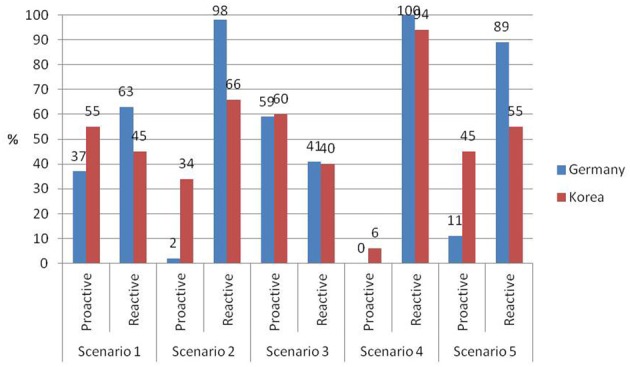
**Relative frequencies of mothers' responses to forced-choice questions of the Caregiver Sensitivity Interview (*n* Korea = 100, *n* Germany = 92)**.

Figure 1 shows relative frequencies of the forced choice answers. The analysis for Scenario One yielded a significant overall model, *R*^2^ = 0.07, χ^2^(4, *N* = 192) = 9.58, *p* < 0.05. Culture was significantly associated with mothers' sensitivity. To be more specific, Korean mothers were less likely to report reactive sensitivity in comparison to German mothers. Further, a marginally significant effect for number of children was found. The probability of using reactive sensitivity was higher the more children a mother had (see Table 1).

**Table 1:** **Summary of binary logistic regression predicting sensitivity in Scenario One**.

**Predictors**	**β**	***SE***	***Odds-Ratio***	***Wald***
Culture	−1.05	0.39	0.35	7.43^**^
Mothers' age	−0.01	0.04	0.99	0.09
Number of children	−0.05	0.18	0.95	0.08
Working status	0.58	0.33	1.78	3.03^+^

+ *p < 0.10*,

** p < 0.01. Proactive sensitivity (always observe a child carefully) = 0; reactive sensitivity (wait until the child requests help) = 1, reference group = Germany.

Regarding Scenario Two the analysis revealed a significant overall model, *R*^2^ = 0.35, χ^2^(4, *N* = 192) = 46.38, *p* < 0.001 and a significant cultural difference with regard to the preference for reactive sensitivity. The majorities of both the German (98%) and the Korean mothers (66%) chose the reactive answer (wait and see what happens). However, Korean mothers were less likely to choose reactive sensitivity in Scenario Two than German mothers. Moreover, the effects of mothers' age and occupational status on the preference for reactive sensitivity were marginally significant. Working mothers had a marginally higher probability of choosing reactive sensitivity than mothers who were not employed. The older the mothers were the higher was the probability of preferring reactive sensitivity (see Table 2).

**Table 2:** **Summary of binary logistic regression predicting sensitivity in Scenario Two**.

**Predictors**	**β**	***SE***	**Odds-Ratio**	**Wald**
Culture	−2.33	0.79	0.10	8.60^**^
Mothers' age	0.13	0.07	1.14	3.82^+^
Number of children	0.71	0.42	2.03	2.85^+^
Working status	−0.56	0.46	0.54	1.43

+ p < 0.10,

** p < 0.01. Proactive sensitivity (come and comfort) = 0; reactive sensitivity (wait until the child starts crying) = 1, reference group = Germany.

For Scenario Three the overall model was not significant, *R*^2^ = 0.01, χ^2^(4, *N* = 192) = 2.07, *p* = 0.723. About 40% of the German and the Korean participants reported that they would let their children know that they can approach the mother when they feel they want to be with her (reactive sensitivity). About two thirds of German and Korean mothers said that they would go and sit close to the child and talk to him or her (proactive response). As Table 3 shows, neither culture nor the control variables were significantly associated with maternal sensitivity.

**Table 3:** **Summary of binary logistic regression predicting sensitivity in Scenario Three**.

**Predictors**	**β**	***SE***	***Odds-Ratio***	**Wald**
Culture	−0.08	0.37	0.93	0.04
Mothers' age	−0.03	0.04	0.97	0.49
Number of children	−0.09	0.18	0.91	0.25
Working status	−0.36	0.32	0.27	1.22

Proactive sensitivity (sit close and talk) = 0; reactive sensitivity (let the child know he/she can approach) = 1, reference group = Germany.

For Scenario Four the full model was marginally significant *R*^2^ = 0.19, χ ^2^(4, *N* = 192) = 8.93, *p* < 0.07. The majorities of German (100%) and Korean mothers (94%) chose the reactive response (child should ask for help) over the proactive one (child should wait for mother to ask). None of the investigated variables was significantly associated with mothers' choice of sensitivity (see Table 4).

**Table 4:** **Summary of binary logistic regression predicting sensitivity in Scenario Four**.

**Predictors**	**β**	***SE***	**Odds-Ratio**	**Wald**
Culture	−18.11	4134.81	0.00	0.00
Mothers' age	0.02	0.13	1.02	0.02
Number of children	−0.19	0.81	0.83	0.05
Working status	−0.92	1.12	0.40	0.68

Proactive sensitivity (child should wait for mother) = 0; reactive sensitivity (child should ask for help) = 1, reference group = Germany.

Regarding Scenario Five logistic regression analysis revealed a highly significant model, *R*^2^ = 0.21, χ^2^(4, *N* = 192) = 30.52, *p* < 0.001. Half of the Korean mothers (45%) chose the proactive response (anticipate children's needs), whereas the majority of German participants (89%) reported that a mother should attend to a child's requests (reactive sensitivity). German and Korean mothers differed significantly regarding their preference for maternal sensitivity (see Table 5). German mothers had a higher probability of choosing reactive sensitivity with regard to Scenario Five than Korean mothers.

**Table 5:** **Summary of binary logistic regression predicting sensitivity in Scenario Five**.

**Predictors**	**β**	***SE***	**Odds-Ratio**	**Wald**
Culture	−2.21	0.50	0.11	19.29^***^
Mothers' age	−0.05	0.05	0.95	1.13
Number of children	−0.11	0.23	0.90	0.22
Working status	−0.01	0.37	0.99	0.00

*** p < 0.001. Proactive sensitivity (anticipate child's needs) = 0; reactive sensitivity (attend to child's explicit requests) = 1, reference group = Germany.

### Parenting beliefs

To provide an accurate overview of the most important results, we integrated the five most frequently verbalized parenting beliefs for proactive and reactive sensitivity across both cultures into this research article.

#### Reactive sensitivity

**Figure 2:**
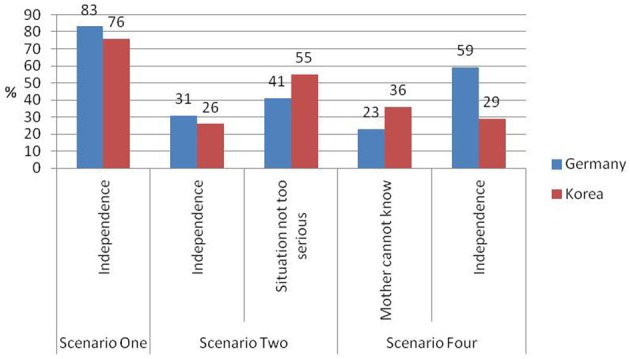
**Relative frequencies of five most frequent parenting beliefs related to reactive sensitivity (*n* Korea = 100, *n* Germany = 92)**.

For reactive sensitivity, the five most frequently stated parenting beliefs and their frequencies across the German and Korean samples are shown in Figure 2.

The binary logistic regression analysis for mothers who preferred reactive sensitivity in Scenario One [overall model was marginally significant *R*^2^ = 0.06, χ^2^(4, *N* = 192) = 8.06, *p* < 0.10] revealed that German mothers were more likely to report that they would wait until the child requested help in order to support development of independence. In Scenario Two, the overall model was not significant *R*^2^ = 0.06, χ^2^(4, *N* = 192) = 7.58, *p* = 0.108, here, German and Korean mothers were equally likely to report that they would rather wait and see what happens, because it is better for the child to cope with the problem independently. Similarly, both German and Korean mothers reported that they would not approach the child because the situation was not too serious. Overall model for this category was not significant *R*^2^ = 0.03, χ ^2^(4, *N* = 192) = 4.35, *p* = 0.361. Here, a marginally significant effect of mothers' age and their response to the open ended question occurred. The older the mothers were the more likely they were to conclude that the situation was not too serious. However, in Scenario Four, Korean mothers were more likely than German mothers to argue that children should ask for help because a mother cannot always be aware of a child's needs, resulting in a marginally significant effect of culture, while the overall model was not significant *R*^2^ = 0.03, χ^2^(4, *N* = 192) = 4.23, *p* = 0.376. In contrast, a significantly higher percentage of German mothers than Korean mothers reported that they would not intervene because they wanted to encourage children's independence. A highly significant overall model was revealed *R*^2^ = 0.15, χ^2^(4, *N* = 192) = 21.96, *p* < 0.001 (see Table 6).

**Table 6:** **Summary of binary logistic regression predicting parenting beliefs related to reactive sensitivity**.

**Predictors**	**β**	***SE***	**Odds-Ratio**	**Wald**
**SCENARIO ONE**				
**Children need to learn what to do by themselves or deal with problems independently (not depending on others)**				
Culture	−1.04	0.38	0.35	7.33^**^
Mothers' age	−0.03	0.04	0.97	0.75
Number of children	−0.04	0.18	0.96	0.06
Working status	0.28	0.33	1.33	0.74
**SCENARIO TWO**				
**It is better for children to solve the problem on their own, it is better to be independent**				
Culture	−0.33	0.44	0.72	0.56
Mothers' age	0.06	0.04	1.06	1.57
Number of children	0.12	0.19	1.12	0.36
Working status	−0.34	0.38	0.72	0.79
**The situation is not too serious and so the child can handle it by her/himself (does not need any help)**				
Culture	0.15	0.38	1.16	0.15
Mothers' age	0.07	0.04	1.07	3.13^+^
Number of children	−0.14	0.18	0.87	0.55
Working status	−0.15	0.33	0.86	0.21
**SCENARIO FOUR**				
**The mother cannot know everything children need; it is difficult for the mother to decide whether to help the child or not**				
Culture	0.69	0.41	1.99	2.76^+^
Mothers' age	−0.01	0.04	0.99	0.08
Number of children	0.11	0.20	1.12	0.32
Working status	−0.34	0.35	0.71	0.95
**It is important for children to do it by themselves and make their own decision; in order to encourage children's independence**				
Culture	−1.09	0.38	0.34	8.01^**^
Mothers' age	0.04	0.04	1.04	0.94
Number of children	0.18	0.19	1.19	0.88
Working status	−0.06	0.34	0.94	0.03

+ p < 0.10,

** p < 0.01. Category did not occur = 0, category occurred = 1, reference group = Germany.

#### Proactive sensitivity

**Figure 3:**
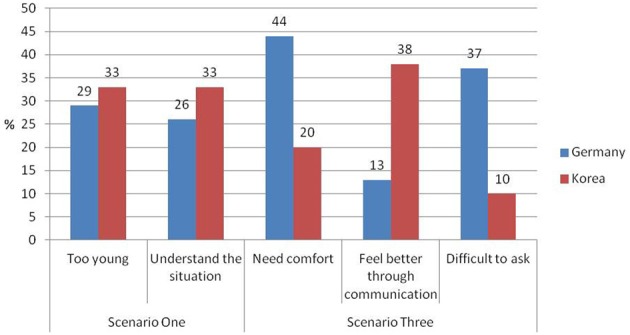
**Relative frequencies of five most frequent parenting beliefs related to proactive sensitivity (*n* Korea = 100, *n* Germany = 92)**.

The five most frequent reasons for proactive behavior and their frequencies can be found in Figure 3.

For Scenario One, there was no significant difference between Korean and German mothers regarding the statement that mothers should always observe their children carefully because children are too young to know what to do. However, the overall model for that category was significant *R*^2^ = 0.09, χ^2^(4, *N* = 192) = 10.12, *p* < 0.05. Further, there was a negative and marginally significant association between the number of children and mothers tendency to refer to children's age in order to explain their behavior. The more children a mother had the less likely she was to report this statement. Moreover, in Scenario One Korean mothers were marginally more likely than German mothers to report that they aim to understand the situation before deciding whether to provide help or not. Yet the overall model was not significant *R*^2^ = 0.05, χ^2^(4, *N* = 192) = 5.72, *p* = 0.221. For Scenario Three regression analyses revealed significant cultural differences regarding mothers most frequent explanation for preferring proactive sensitivity. German in comparison to Korean mothers more frequently explained that they would sit close to an upset child in order to provide comfort. However, the overall model was not significant *R*^2^ = 0.06, χ^2^(4, *N* = 192) = 7.31, *p* = 0.120. Furthermore, German mothers were more likely than Korean mothers to report that children are not able to express their need for help, *R*^2^ = 0.11, χ^2^(4, *N* = 192) = 12.20, *p* < 0.05. On the other hand, more Korean mothers than German mothers would sit next to the child in order to improve the child's mood by communicating with him or her. Analyses revealed a significant overall model for this category *R*^2^ = 0.11, χ^2^(4, *N* = 192) = 12.20, *p* < 0.05 (see Table 7).

**Table 7:** **Summary of binary logistic regression predicting parenting beliefs related to proactive sensitivity**.

**Predictors**	**β**	***SE***	**Odds-Ratio**	**Wald**
**SCENARIO ONE**				
**Children are too young to know what (how) to do or decide if they need help**				
Culture	0.38	0.52	1.46	0.53
Mothers' age	−0.08	0.06	0.93	1.96
Number of children	−0.64	0.34	0.53	3.63^+^
Working status	−0.36	0.45	0.70	0.64
**To understand the situation whether children need help or for the mother's decision to help or not**				
Culture	1.07	0.55	2.92	3.74^+^
Mothers' age	0.01	0.06	1.01	0.05
Number of children	0.32	0.24	1.38	1.74
Working status	−0.01	0.44	0.99	0.00
**SCENARIO THREE**				
**Children need someone who provides comfort or to talk to**				
Culture	−1.02	0.49	0.36	4.34^*^
Mothers' age	0.01	0.05	1.01	0.04
Number of children	0.08	0.21	1.09	0.16
Working status	0.36	0.41	1.43	0.76
**In order to make the child feel better through communication**				
Culture	1.57	0.58	4.80	7.22^**^
Mothers' age	−0.02	0.06	0.98	0.16
Number of children	0.43	0.25	1.53	2.88^+^
Working status	−0.30	0.44	0.74	0.47
**Because it is difficult for children to ask for help first**				
Culture	−1.63	0.59	0.20	7.61^**^
Mothers' age	−0.01	0.05	0.88	0.02
Number of children	−0.22	0.26	0.80	0.77
Working status	0.10	0.48	1.11	0.05

+ p < 0.10.

* p < 0.05,

** p < 0.01. Category did not occur = 0, category occurred = 1, reference group = Germany.

To conclude, German as well as Korean mothers engage in both proactive and reactive sensitivity. In line with previous studies (e.g., Rothbaum et al., 2006), analyses revealed that according to participants' reports, German mothers were more likely to expect that children request support and clearly communicate their needs. In contrast, Korean mothers tended to report that a mother should always observe a child carefully to be able to intervene proactively if necessary. Regarding parenting beliefs, the present analyses revealed that the German and Korean mothers who preferred reactive behavior aimed to encourage children's independence. In contrast, German and Korean mothers explained proactive behavior as attempts to avoid anything negative happening to the child and as helping the child with his or her emotional distress.

## Discussion

The results of the present study have shown that German and Korean mothers differ with regard to their sensitivity. As expected, the majority of the German sample chose the reactive option in almost every forced-choice scenario (except Scenario Three), which is in line with previous findings in Western samples (especially the United States; e.g., Rothbaum et al., 2000, 2006). Mothers stated that they would encourage their children to solve problems on their own and to independently verbalize their needs, presumably aiming to foster children's independence. With regard to the Korean mothers' responses, the findings were less clear. For Scenarios One and Three, Korean mothers preferred the proactive over the reactive response option. This might point to the importance of the mothers guiding and structuring children's behavior (Rothbaum et al., 2000, 2006; Trommsdorff and Rothbaum, 2008). Regarding Scenarios Two, Four, and Five, the majority of Korean participants preferred the reactive over the proactive option. This response pattern may point to a more situation-specific sensitivity for the Korean mothers in comparison to the German mothers. Trommsdorff and Friedlmeier (2010) similarly reported that German mothers' sensitivity was stable across different situations (children's self- and other-focused distress) while Japanese mothers' sensitivity varied according to the situational context.

Further, results suggested that maternal sensitivity in general may depend on the situational context. In Scenario Two, the majority of both German and Korean mothers decided not to go to the child (reactive sensitivity), indicating that they perceived the situation as not too serious and that the child should handle it alone. On the other hand, in Scenario Three in which the child is obviously unhappy, the majority of German and Korean mothers preferred to approach to the child (proactive sensitivity) indicating the mothers' high responsiveness to children dealing with negative emotional feelings.

Concerning the parenting beliefs related to the preferences of pro- or reactive sensitivity, we found both cross-cultural differences and similarities. For the preference of proactive sensitivity, German and Korean mothers did not differ in their opinion that proactive behavior is necessary according to children's developmental stage. However, results revealed that Korean mothers were marginally more likely to reason that they want to understand the situation (Scenario One). German mothers who decided for the proactive option in Scenario Three said they would choose to sit close to an upset child because the child would need to be comforted and need someone to talk to. This finding points to the German mothers getting engaged in the child's emotions. Also, more German than Korean mothers would attend the child because they believed that it might be difficult for children to ask for help or to express that they need help. These beliefs may indicate that the mothers try to encourage their children to express their feelings, which is important for the development of emotion regulation in Western societies (Trommsdorff and Rothbaum, 2008). On the other hand, the Korean mothers stated that they would approach an upset child to try to cheer the child up through communication. That is, the mother tries to distract the child from the negative emotion. This is in line with the assumption of Rothbaum et al. (2000) that proactive sensitivity focuses on emotional closeness and helping children to cope with negative emotions.

Regarding reactive sensitivity, German mothers explained their choice mainly by attempts to encourage their children's independence. This result indicates the individuality and separateness of mother and child in independence-oriented cultures. Korean mothers who preferred reactive sensitivity also aimed to encourage children's independence. Moreover, they claimed that a mother is not able to know everything a child needs or whether she should help the child or not. Therefore, Korean mothers who chose reactive sensitivity seem to indicate that it is difficult to anticipate children's needs.

Although mothers' working status was not significantly associated with their choice for pro- or reactive sensitivity there is reason to assume that mothers who work outside the home prefer reactive sensitivity because they cannot stay with the child the whole day. According to the literature, contemporary Korean socialization beliefs appear to be influenced by Western ideas regarding socialization while traditional values and beliefs are still preserved (Park and Cheah, 2005; Chang and Song, 2010). Schwarz et al. (2005) found that the parenting goal of independence in a German sample was primarily related to individualistic values, while in the Korean sample this parenting goal was positively associated with both collectivistic and individualistic values. Due to the rising number of working mothers in Korea the child's autonomy may become more important. For a mother who is not always present and thus cannot always observe, it may be more difficult to anticipate what her child wants.

The present study revealed valuable insights about German and Korean mothers' sensitivity and related parenting beliefs. This is the first time that the concepts of pro- and reactive sensitivity and their related parenting beliefs (which are supposed to motivate parenting behaviors like pro- or reactive sensitivity) were studied by investigating mothers of first graders in cross-cultural comparison. Due to the use of both forced-choice items and open-ended questions it was possible not only to figure out the parenting beliefs related to mothers' preferences of sensitivity but also to compare them across two different cultural contexts.

There are also some limitations that have to be addressed. First, mothers were interviewed face to face which might have evoked social desirability. Also, data were subjective (maternal self-reports) and not observational. Moreover, the investigated scenarios describe very different situations typical for every day mother-child interactions. It would be helpful to generate more situations and also scales to have similar scenarios for different domains for investigating patterns of maternal sensitivity more precisely. In addition, it could be helpful to include the same scenarios for assessing normative and non-normative evaluation of the mothers' but also from the children's point of view. Further, both the Korean and the German participants were more highly educated than the general population of the two countries. Hence, the samples are not representative and generalization of results is not indicated. Besides these limitations, the study design avoids disadvantages which usually affect quantitative cross-cultural comparisons, for example bias due to culture specific response tendencies (van de Vijver and Leung, 1997). Further, by having the mothers elaborate their opinions culture specificities can be detected which might have been overseen by using scales.

Further research should use multi-method assessments including behavior observations in order to draw a more distinct picture of maternal sensitivity. In addition, longitudinal studies could be used to detect if different kinds of maternal sensitivity may be linked to children's different developmental outcomes in different cultural contexts. It might also be informative to extend the study to the diverse caregivers (e.g., fathers, grandparents) who may influence children's development. Also, the role of the child as an active agent in the socialization process should not be neglected.

Parental intuitive theories consist of a great number of variables in addition to parenting behavior (e.g., parenting goals, emotions, attributions) as well as the relations between these concepts (Trommsdorff et al., 2012). The present study contributes to the understanding of parental intuitive theories in cultural context by drawing a clearer picture about maternal sensitivity and related beliefs as one aspect of parenting behavior in different cultural settings. The study is conducive to the notion that cross-cultural comparisons should not be based on dichotomous concepts. Mothers from both cultures showed proactive as well as reactive sensitivity, partly according to the situational context. Further, mothers' beliefs related to reactive and proactive sensitivity almost did not differ in the two cultural contexts. Mothers decided for reactive sensitivity in order to support children's independence. Mothers who preferred proactive sensitivity did so because of the child's immaturity or to help the child with emotional distress. However, the degree of mothers' use of proactive and reactive sensitivity was found to be culturally diverse. This finding might be related to different cultural values that are based on historical backgrounds but also to current ongoing change. Further, cultural context and social change might not only influence parenting but also psychological health and well-being of parents and children (e.g., conflict between traditional and modern values and practices), so being aware of cross-cultural differences in diverse contexts can contribute to improvements in applied areas (Trommsdorff and Heikamp, 2013), for example detection and treatment of neuro-developmental disorders (Norbury and Sparks, 2013).

### Conflict of interest statement

The authors declare that the research was conducted in the absence of any commercial or financial relationships that could be construed as a potential conflict of interest.
